# SignFormer-GCN: Continuous sign language translation using spatio-temporal graph convolutional networks

**DOI:** 10.1371/journal.pone.0316298

**Published:** 2025-02-14

**Authors:** Safaeid Hossain Arib, Rabeya Akter, Sejuti Rahman, Shafin Rahman

**Affiliations:** 1 Department of Robotics and Mechatronics Engineering, University of Dhaka, Dhaka, Bangladesh; 2 Department of Electrical and Computer Engineering, North South University, Dhaka, Bangladesh; Purdue University, UNITED STATES OF AMERICA

## Abstract

Sign language is a complex visual language system that uses hand gestures, facial expressions, and body movements to convey meaning. It is the primary means of communication for millions of deaf and hard-of-hearing individuals worldwide. Tracking physical actions, such as hand movements and arm orientation, alongside expressive actions, including facial expressions, mouth movements, eye movements, eyebrow gestures, head movements, and body postures, using only RGB features can be limiting due to discrepancies in backgrounds and signers across different datasets. Despite this limitation, most Sign Language Translation (SLT) research relies solely on RGB features. We used keypoint features, and RGB features to capture better the pose and configuration of body parts involved in sign language actions and complement the RGB features. Similarly, most works on SLT research have used transformers, which are good at capturing broader, high-level context and focusing on the most relevant video frames. Still, the inherent graph structure associated with sign language is neglected and fails to capture low-level details. To solve this, we used a joint encoding technique using a transformer and STGCN architecture to capture the context of sign language expressions and spatial and temporal dependencies on skeleton graphs. Our method, SignFormer-GCN, achieves competitive performance in RWTH-PHOENIX-2014T, How2Sign, and BornilDB v1.0 datasets experimentally, showcasing its effectiveness in enhancing translation accuracy through different sign languages. The code is available at the following link: https://github.com/rabeya-akter/SignLanguageTranslation.

## 1 Introduction

Millions of individuals who are deaf or hard of hearing communicate through sign languages worldwide. Sign languages, characterized by their unique differences, richness, and complexity, are similar to spoken languages, and they emphasize the significance of developing SLT technology. SLT converts sign language videos to spoken language to ensure effective communication between those who use sign language and those unfamiliar with sign language. This technology has the potential for real-life applications, such as in educational institutions, workplaces, healthcare facilities, public services, etc. Researchers have developed various methodologies to work on both isolated sign language recognition (SLR), which identifies individual signs, and continuous SLT, which translates full sentences and is needed for natural and effective communication [1–17]. However, the state-of-the-art for continuous SLT is still evolving. In this paper, we propose a new method of continuous SLT, especially targeting low-resource languages like Bangla Sign Language (BdSL).

Sign language users employ two distinct types of signals to convey information: manual elements, encompassing hand movement, arm orientation, etc., and non-manual elements, consisting of facial expressions, mouth actions, eye movements, eyebrow gestures, head movements, body postures, etc. [18, 19]. Using only the RGB feature for tracking these signals and training models can be limiting as discrepancies between the backgrounds and signers in training and testing datasets can lead to a decline in translation efficiency [3]. In contrast, the keypoints feature can capture fine-grained details about the pose and configuration of the body parts involved in the sign language action, which is complementary to the RGB feature. Taking motivation from action recognition tasks [20–22], we utilize both RGB and keypoint features to get a better representation of sign language videos. Early research on sign language primarily focused on recognition, but that was limiting effective communication [19, 23–28]. Then, researchers moved their focus working on sign language translation [1–9]. However, very few studies have utilized both RGB and keypoint features simultaneously for SLT [3]. To our knowledge, this is the first work to employ RGB and keypoint features for sign language translation in a gloss-free context.

Existing state-of-the-art methods on SLT have worked with transformer architecture to solve the sequence-to-sequence problem of SLT [5–9]. Transformer architecture can capture the broader context of sign language sequences as it can effectively capture long-distance dependencies, and its attention mechanism focuses on the most relevant video frames while translating texts from sign language videos. Nevertheless, the transformer architecture neglects the inherent graph structure present in the data, which poses challenges in capturing the fine-grained meanings associated with each sign. Graph structures can offer insight into the inter-relationships among the joint points during sign language actions. Consequently, while Transformers are powerful for high-level context understanding, their limitation in capturing low-level details limits their effectiveness in fully translating the complexities of sign language expressions. To solve the problems, we employ a join encoding technique that fuses transformer architecture with Spatial-Temporal Graph Convolutional Networks (STGCN) to get a representation of the sign language videos in both broader and local fine-grained contexts. Moreover, STGCN efficiently learns spatial and temporal dependencies on skeleton graphs.

One major challenge in developing the SLT model is the lack of large and diverse datasets for training. We worked on three sign language datasets: RWTH-PHOENIX-2014T [2], How2Sign [29], and BornilDB v1.0 [30]. Notably, the BornilDB v1.0 [30] dataset presents significant challenges, including only three signers (two males and one female). While developing a model for such low resource SLT, the model tends to learn characteristics of particular individuals, introducing a significant level of bias to the model and thus limiting real-world applications. Additionally, the BornilDB v1.0 [30] dataset is particularly challenging than the previous three datasets because of the varying camera quality, dynamic backgrounds, and inconsistent lighting conditions across videos. To address these challenges in the datasets and to make our model applicable for real-world applications beyond our evaluation datasets, we included both key points and RGB features in our pipeline. We got comparably good results in RWTH-PHOENIX-2014T [2] and How2Sign [29] dataset, and a baseline performance for BornilDB v1.0 [30] dataset. These results demonstrate the effectiveness of our model in the translation of different sign languages while guiding future research direction for continuous low-resource sign language translation. The primary contributions of the paper can be outlined as:

- **Architectural Fusion:** We integrate both transformer and STGCN architecture to enhance our method’s, **SignFormer-GCN**, ability to extract meaningful representation by leveraging contextual and spatio-temporal information at both broader and fine-grained levels.
- **Fusion Strategy:** We explore different fusion strategies between these architectures to identify the most effective one.
- **State-of-the-Art Performance:** SignFormer-GCN provides new state-of-the-art performance of sign language translation in How2Sign [31] and BornilDB v1.0 dataset [30] to guide future research in continuous sign language translation.

The rest of the paper is organized as follows: in Section 2, we survey the previous studies and state-of-the-art on SLT. Then, we introduce our methodology in Section 3. We share our experimental setup and evaluation protocol in the subsequent section. Following this, we report our methodology’s quantitative and qualitative results and include a detailed ablation study in Section 4. Finally, we conclude the paper by discussing our findings and the future direction of our work in Section 5.

## 2 Related work

### 2.1 Sign language recognition

Initial research on SLR primarily focused on isolated SLR [19, 23–28, 32], which identifies individual signs but lacks the natural flow of sign language communication. Limitations of isolated SLR spurred the development of continuous SLR [10–17], that aims to continuously recognize sign gestures, aligning with sign language grammar and structure and catering to the preferences of sign language users.

### 2.2 Sign language translation

In pursuit of effective communication between sign language users and those unfamiliar with sign language, extensive research has been done on continuous SLT. Initially, SLT employed Recurrent Neural Networks (RNNs) [33] within the encoder-decoder framework, utilizing either Gated Recurrent Units (GRUs) or Long Short-Term Memory (LSTM) [7, 34–36]. However, addressing the limitations of RNNs in handling long-term dependencies has led to adopting more effective attention-based methods. The transformer network [37], known for its success in various domains [38–43], derives its efficacy from its self-attention mechanism. This feature has been found favourably in recent SLT research, leading to the widespread adoption of transformer architecture. However, transformers can only capture and learn contextual information and patterns. It cannot take advantage of the inherent graph structure of joints in a human body. Graph-based methods have shown significant success in human activity recognition by effectively modeling joint relationships. Spatial-temporal graph convolutional networks’ (STGCN) potential to learn spatio-temporal dynamics makes them highly effective at learning and modeling structured patterns from motion sequences. [44, 45]. Consequently, this architecture has been employed to capture spatial and temporal relationships that are essential for SLR [46, 47]. However, a fusion of contextual and spatio-temporal relationships using transformer and STGCN architecture has not been explored, which overlooks important aspects of sign language. In our method, we leveraged both these architectures to get a better and more meaningful representation of the sign gestures.

### 2.3 Sign language translation based on gloss supervision

SLT based on gloss supervision categorizes current SLT methods into two-stage gloss-supervised methods, end-to-end gloss-supervised methods, and end-to-end gloss-free methods. Gloss, a textual representation of sign gestures in spoken or written language, serves as an intermediary in the first two approaches [1–4]. However, acquiring gloss annotations can be costly as it requires the expertise of sign language professionals. In contrast, gloss-free methods do not rely on intermediary gloss annotations, directly translating sign language videos into spoken language texts [5–9]. Our method belongs to the gloss-free category as we utilize a direct translation from sign language video to spoken language text without any involvement of gloss annotation.

### 2.4 Tokenization methods of sign language videos

Various SLT approaches have employed different tokenization methods for sign language videos. Some utilized 2D CNN features extracted from video frames at the gloss-level [1, 2]. Inflated 3D convnets (I3D), initially designed for action recognition [48], have been further trained using sign language data [7, 8, 49–52]. S3D [53] features have been employed after pretraining with the WLASL dataset and kinetics [54]. Additionally, some approaches have used pose estimators [55, 56] to represent video sequences, as they provide information on the position and movement of body parts [6, 35, 36, 57]. Lastly, some methods combined video and keypoint features to capture more meaningful representation [3, 58]. Our method aligns with this last-mentioned method as we use both RGB video encoding and keypoint encoding.

## 3 Methodology

In this section, we introduce SignFormer-GCN, which is an end-to-end model that learns to translate sign language video sequences into spoken language directly.

**Problem Formulation:** Given a sign language video *V*_*i*_ = {*f*_1_, *f*_2_, …, *f*_*t*_} with *t* frames, our goal is to learn the conditional probabilities *P*(*S*_*i*_|*V*_*i*_) of generating a translated sentence *S*_*i*_ = {*w*_1_, *w*_2_, …, *w*_*n*_} with *n* words. The training dataset consists of a set of tuples {(*V*_*i*_, *S*_*i*_):*i* ∈ [1, *M*]} where *M* represents the total number of training videos.

Solving the SLT problem presents quite a few challenges. Sign gestures can vary in length because different signers do them at different paces. Besides, as each video frame does not map one to one with each of the tokens of translated sentences, the detailed meaning expressed through sign gestures might include subtle local aspects because of differences in grammatical rules and ordering in sign languages and spoken languages. Because of transformer networks’ effectiveness in modelling sequence-to-sequence tasks, recent methods on SLT employed this architecture [2]. However, it is important to recognize that the representation found in this architecture has its limitations. While transformer architecture excels at learning contextual relationships, this architecture struggles to capture the topological aspect of the joints of a human body, which is equally important in sign language.

**Solution strategy:** Sign language gestures that involve movements of human body joints are best represented through a spatio-temporal skeleton graph. We can use such a graph to learn a new set of feature representations. To bridge the limitation of transformer architecture, we employ Spatio-Temporal Graph Convolutional Networks (STGCN) to extract the relationship between spatial and temporal features from the skeletal structure. Using only one type of feature representation from the architecture is limiting as they can give both contextual and spatio-temporal information together. To make the translation task more efficient, we incorporated transformer and STGCN architecture in our method to learn a better and more meaningful representation.

### 3.1 Model overview

**Fig 1:**
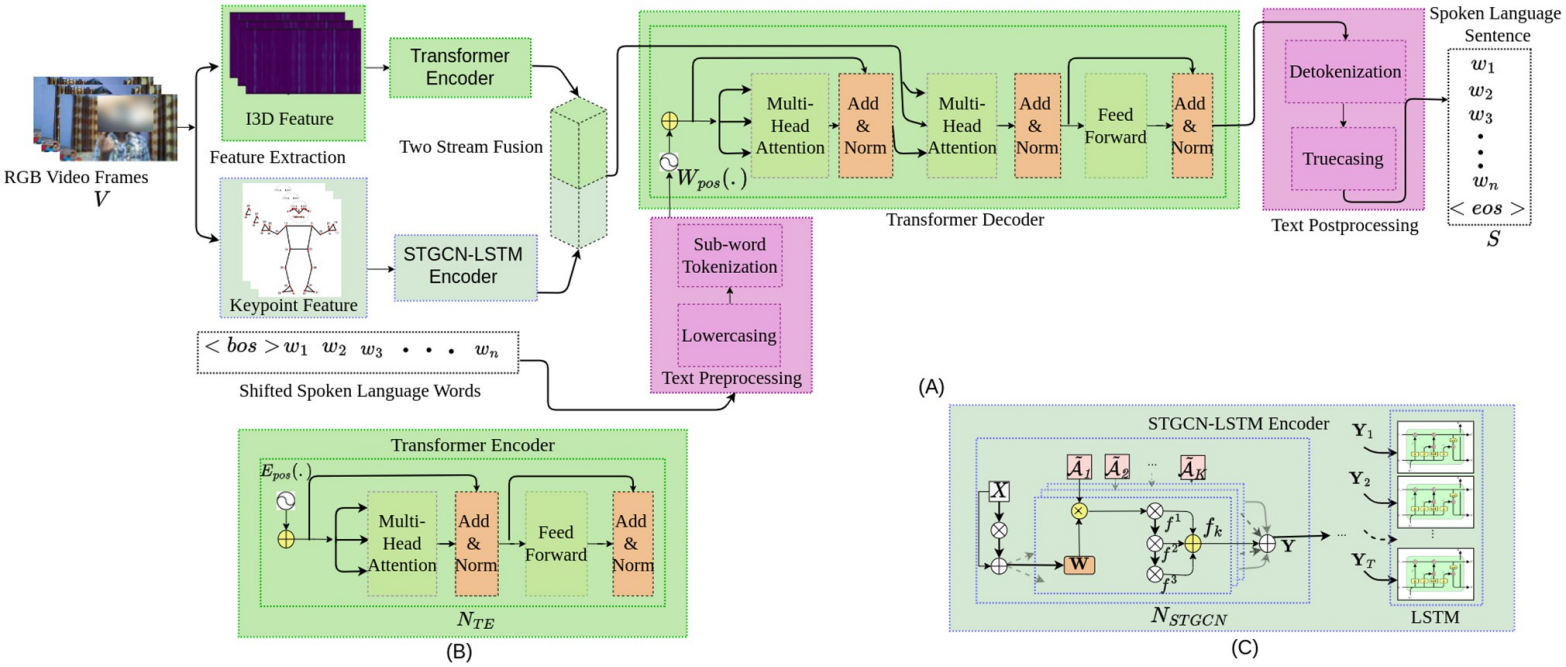
Overview of the SignFormer-GCN methodology for sign language translation. (A)An overview of our two-stream fusion methodology, SignFormer-GCN, for sign language translation. The methodology fused the RGB feature processed by the transformer encoder, and the STGCN-LSTM encoder processed the keypoint feature, and the fused output was passed to a transformer decoder for final translation. (B) An overview of the transformer encoder. (C) An overview of STGCN-LSTM encoder.

An overview of our model is shown in Fig 1. We fuse two stream encoding processes to encode the sign language videos, $V\in R^{T\times N\times C}$. In the first stream, i3d features, $E_{t}\in R^{T\times C}$, are extracted using an i3d network. Then, this feature is positionally encoded before being fed into a transformer encoder that is formed with several transformer encoder blocks, *N*_*TE*_. The ultimate output encoding of this stream is denoted as $Z_{t}\in R^{T\times C}$. In the second stream, we extract keypoint features using the Mediapipe algorithm. This process establishes a spatio-temporal graph structure, *G* = (*V*, *E*, *A*), incorporating *K* joints and *T* frames. Then, the keypoint data is passed through an STGCN-LSTM Encoder formed by multiple STGCN blocks, *N*_*STGCN*_, followed by an LSTM layer. This stream finally gives an encoding represented as $Y\in R^{T\times N\times (C_{out})}$. These two streams are fused in the next step to pass the final encoding to a transformer decoder formed with multiple transformer decoder blocks, *N*_*TD*_. The preprocessed text is also passed to the transformer decoder. The decoder generates tokenized output, which is detokenized in the post-processing step to generate the predicted spoken language text, $S\in R^{N}$. Commonly used notations are summarized in Table 1 for clarity.

**Table 1:** Commonly used notations.

Notations	Descriptions
*F*_temporal(.)_	Temporal aggregation function
*P*(.)	Projection operation
$E_{t}\in R^{C}$	Feature embedding of the video
$\hat{\mu }\in R^{C}$	Embedded representation of the text
*E*_*pos*_(.)	Positional encoding function for the text
*G*	Graph.
V	Set of nodes in a graph, *V* = {*v*_*tj*_|*t* = 1, …, *T*;*j* = 1, …, *J*}
E	Set of edges in a graph, *E* = {*v*_*tj*_*v*_*tk*_ ∪ *v*_*tj*_*v*_(*t*+1)*j*_ ∣ *t* = 1, …, *T*, (*j*, *k*) ∈ *S*}
A	Adjacency matrix
$A_{k}$	*k*th adjacency matrix
I	Identity matrix
*D*_*k*_	*k*th diagonal degree matrix
*N*	Number of nodes, N=|V|
*J*	Number of joints
*T*	Number of frames
*S*	Connectivity of human skeleton structure
*l*	The layer index
*t*	The time step index
⊗	Temporal convolution operation.
⊕	Concatenation operation
*C*_*m*_	Number of input channels
*C*_*n*_	Number of output channels of STGCN
*C*_*o*_	Number of output channels of LSTM
$X\in R^{T\times N\times C_{m}}$	Input feature matrix of STGCN
$Y^{l}\in R^{T\times N\times C_{n}}$	Output from the STGCN block
$\Gamma_{i}^{l}$	The *i*^*th*^ temporal kernal of *l*^*th*^ STGCN block
$Y_{r}\in R^{N\times NC_{n}}$	Input feature of LSTM
*σ*_*g*_(⋅)	Sigmoid activation function
*σ*_*c*_(⋅)	Tanh activation function.
*W*_*i*_, *W*_*o*_, *W*_*f*_, *W*_*c*_	Weight matrices for the input features of LSTM
*U*_*i*_, *U*_*o*_, *U*_*f*_, *U*_*c*_	Weight matrices for the hidden states of LSTM
$b_{f},b_{i},b_{o},b_{c}\in R^{C_{m}}$	Bias vectors of LSTM

### 3.2 Joint encoding

#### Video encoding

We employ an I3D network [59] over 16-frame video clips, *F*_I3D_(*V*_*t*:*t*+15_), to give an embedding, *ψ*_*v*_. This number of frames is taken because of its effectiveness in sign language recognition methods [24, 49]. Then, we temporarily aggregate the *ψ*_*v*_ to a vector of constant size. Finally, the vector is transformed into the *C* dimensional embedding space.

$$\begin{array}{c} E_{t}=P\left(F_{\text{temporal}}\left(F_{\text{I3D}}\left(V_{t:t+15}\right)\right)\right) \end{array}$$ (1)

Here, $E_{t}\in R^{C}$ is the feature embedding of the video, *F*_temporal(.)_ is the temporal aggregration function and *P*(.) is the projection operation. As for translating sign language, the position of sign gestures in the entire video sequence is essential information. However, transformer networks do not have access to positional information. To overcome this limitation, we introduce temporal ordering information to the embedded representations of the video using positional encoding *E*_*pos*_(.).

$$\begin{array}{c} \hat{E_{t}}=E_{t}+E_{pos}\left(t\right) \end{array}$$ (2)

We train a transformer encoder model using the positional encoded embedding of the video frames, ${\hat{E}}_{1:T}$. Initially, the embedding of the video frames is passed to a self-attention layer, which is responsible for learning the contextual connections among the embedding of the video frames. Then, the results of the self-attention layers are forwarded through a non-linear point-wise feed-forward layer. Throughout the process, residual connections and normalization are applied for effective training. The process can be formulated using the following equation, where *Z*_*t*_ is the spatiotemporal representation of the frame *f*_*t*_ at time step *t*, given the embedding of all the frames of the video, ${\hat{E}}_{1:T}$. Multiple encoder blocks are stacked to extract the features.

$$\begin{array}{c} Z_{t}=\text{TransformerEncoder}\left({\hat{E}}_{t}|{\hat{E}}_{1:T}\right) \end{array}$$ (3)

#### Keypoint encoding

We employ Mediapipe [56] algorithm for extracting keypoint features. Then, we construct an undirected spatiotemporal graph structure *G* = (*V*, *E*, *A*) with *J* joints and *T* frames, comprising both intra-body and frame-by-frame connections. The set of vertices *V* = {*v*_*tj*_|*t* = 1, …, *T*;*j* = 1, …, *J*} includes all the joints in the sequence of frames. The feature vectors of each vertex *v*_*tj*_ will be 3D coordinate vectors represented by *M*_*g*_(*v*_*tj*_) = {*x*, *y*, *z*}, where *x*, *y*, and *z* are coordinates along each axis in a 3D coordinate space. The joints are connected with edges, *E* = {*v*_*tj*_*v*_*tk*_∪*v*_*tj*_*v*_(*t*+1)*j*_ ∣ *t* = 1, …, *T*, (*j*, *k*) ∈ *S*}, according to the connectivity of the human skeletal structure, *S*, for a frame, and each of the joints connection to itself through consecutive frames. $A\in R^{N\times N}$ is the adjacency matrix of the graph *G*. The adjacency matrix element is 0 if there is no connection between joints and one if there is a connection between joints.

We pass the keypoint data of a video, $X\in R^{T\times N\times C_{m}}$, to STGCN block to extract features from the body-joints. Here, *T* denotes the temporal length; *N* denotes the number of skeleton joints; *C*_*m*_ denotes the number of input channels. This at first extracts the spatial features, $Z\in R^{T\times N\times (C_{m}+C_{filter})}$, by performing temporal convolution to the input keypoint sequences using kernel, Γ and concatenating with input to extract spatial features. Here, *C*_*filter*_ is the number of filters employed in temporal convolution.

$$\begin{array}{c} Z=X⊕(\Gamma ⊗X) \end{array}$$ (4)

Here, ⊗ and ⊕ denote the temporal convolution and concatenation respectively. We perform graph convolution on **Z** at time *t* using the layer, *l*, wise update rule of GCN [60].

$$\begin{array}{c} Z_{t}^{l+1}=\sigma \left(\sum_{k=0}^{K}{\tilde{D_{k}}}^{-\frac{1}{2}}\left(A_{k}+I\right)D_{k}^{-\frac{1}{2}}Z_{t}^{l}{W_{k}}^{l}\right) \end{array}$$ (5)

Here, *k* is the number of level of aggregation, **A**_*k*_ is the *k*th adjacency matrix, **I** is the identity matrix, **D**_*k*_ is a diagonal degree matrix, **W**_*k*_ is the learnable weight matrix and *σ* is an activation function.

Then, three layers of Temporal Convolution Layers (TCNs) with the same padding and kernel Γ^1^, Γ^2^, and Γ^3^, respectively, were implemented to obtain temporal features at various levels. We combined both low-level and high-level features to identify movement patterns while performing a gesture in sign language at various levels of complexity. The operations can be described as the following equations.

Here, spatiotemporal features extracted from the *k*th layer are represented by *f*_*k*_. Lastly, the output from each layer is concatenated to get the final output.

$$\begin{array}{c} Y=f_{1}⊕f_{2}⊕\ldots ⊕f_{k} \end{array}$$ (6)

The result of the STGCN block is denoted as $Y\in R^{T\times N\times C_{\text{n}}}$. Here, $C_{n}=\sum_{i=1}^{k}C_{l}^{i}$ refers to a tensors of three dimensions. We stack multiple STGCN blocks in our method to extract complex spatio-temporal features. After that, we pass the output of the last STGCN block, *Y*^*l*^, to the LSTM block. The LSTM block captures the sequential dependencies in spatio-temporal feature vectors. We used the LSTM block after the STGCN blocks because of its ability to consider the change of spatiotemporal features along the temporal dimension. LSTM is very helpful in the processing of variable-length sequences. In sign language, the same sign done by different signers can have different lengths because it is done at different paces and in different situations. For this, LSTM is a better fit for this sequence problem solution.

For this, *Y*^*l*^ were reshaped to $Y^{r}\in R^{T\times NC_{n}}$, which is the input to the LSTM. LSTM involves the following operations:

Here, *i*^*t*^, *f*_*t*_ and $o_{t}\in R^{C_{m}\times C_{o}}$ denote the input gate, forget gate and output gate respectively. *C*_*o*_ is the number of units in an LSTM cell. **W**_*f*_, **W**_*i*_, **W**_*o*_ and $W_{c}\in R^{C_{m}\times NC_{n}}$ are the weight matrices, **U**_*f*_, **U**_*i*_, **U**_*o*_ and $U_{c}\in R^{C_{m}\times C_{m}}$ are the weight matrices for the preceding hidden state, *b*_*f*_, *b*_*i*_, *b*_*o*_ and $b_{c}\in R^{C_{m}}$ are four bias vectors, *σ*_*g*_ and *σ*_*c*_ are the sigmoid and tanh activation functions. The cell state and the hidden states are denoted by *C*^*t*^ and $h^{t}\in R^{C_{m}\times C_{o}}$ respectively.

#### Two-stream fusion module

The fusion process integrates the I3D feature obtained from the transformer encoder, denoted as *Z*_*t*_, and the encoding from the STGCN-LSTM architecture, denoted as *L*_*t*_. The fused encoding, denoted as Fused Encoding, is mathematically represented as:

$$\begin{array}{c} \text{Fused}\;\text{Encoding}=Z_{t}+L_{t} \end{array}$$ (7)

### 3.3 Training pipeline

#### Text encoding

For text encoding, we used Sentencepiece tokenizer [61], which segments the text into sub-word units. Splitting longer sentences into sub-word units enables us to learn better representations of phonetic variants and compound words. This approach is beneficial for acquiring less common words and addresses issues related to out-of-vocabulary words. After tokenization, positional encoding is done to have temporal information of the text. Following is the formulation of this process, where $\hat{\mu }\in R^{C}$ is the embedded representation of the text, and *E*_*pos*_(.) is the positional encoding function for the text.

$$\begin{array}{c} \hat{\mu }=\text{SubWordEmbedding}\left(w_{n}\right)+W_{pos}\left(n\right) \end{array}$$ (8)

#### Decoding output

At the beginning of the target spoken language sentence, we add a special token <*bos*>. Then, position-encoded word embedding is fed to a masked self-attention layer. Masking ensures that each token only considers its preceding tokens while gathering contextual information. Marking is necessary as the decoder cannot access future output tokens during inference. The combined video and text embedding are passed to the decoder layer to learn the reference and target sequence mapping. The transformer decoder is trained to generate target words sequentially until it reaches the <*eos*> token. The decoding process is formulated as follows:

$$\begin{array}{c} H_{n+1}=\text{TransformerDecoder}\left({\hat{\mu }}_{u}|{\hat{\mu }}_{1:n-1},Z_{1:T}\right) \end{array}$$ (9)

The overall probability of the sentence, *p*(*S*|*V*) is calculated by multiplying the probabilities of individual words given their respective contexts, *H*_*u*_. This can be formulated as:

$$\begin{array}{c} p\left(S|V\right)=\prod_{u=1}^{n}p\left(w_{u}|H_{u}\right) \end{array}$$ (10)

#### Loss optimization

We employed Labeled Smoothed Cross Entropy ($L_{CE}$) loss to train our method [31, 62, 63]. This loss is a modified version of cross entropy with the integration of label smoothing, encouraging the model to generalize translation to unseen translation. This loss function clusters the representations for training examples of the same class. $L_{CE}$ loss can be formulated by the following equation, where *ce*(*x*) denotes the standard cross-entropy loss of *x*, *ϵ* is a small positive number, *y* is the correct class, $\hat{y}$ is the incorrect class, and *N* is the number of classes.

$$\begin{array}{c} L_{CE}=\left(1-\epsilon \right)ce\left(y\right)+\epsilon \sum \frac{ce(\hat{y})}{N} \end{array}$$ (11)

### 3.4 Analysis

Most works in SLT utilize transformer architecture to address the problem [31]. While transformer architecture excels at sequence-based tasks like SLT due to its ability to understand broader context and long-range dependencies, it often overlooks the inherent graph structure in sign language videos. In their experiment, [31] observed that when working with poses, the decoder tends to disregard the conditioning provided by the encoder and operate primarily as a language model. This behaviour could be attributed to feeding poses as sequences of one-dimensional arrays containing only landmark coordinates. Consequently, they excluded keypoint features from their architecture, ignoring pose’s graph-like structure. Our approach, however, incorporates additional streams of keypoint features using an STGCN-LSTM encoder alongside the RGB feature and transformer architecture stream. This allows our model to benefit from a richer input representation, capturing both visual and pose-related details. As a result, this enhancement can improve the model’s understanding of sign language videos and potentially increase translation quality.

Most of the works in SLT that utilized graph-based methods focus on SLR task [64–68]. Among the few works that address SLT using graph-based methods, they incorporate gloss annotation in their training pipeline [69].

Among the works in SLT that utilize both RGB and keypoint features, Chen et al. [3] employ gloss annotation to train their model, which is restricting due to the expensive nature of gloss annotation. In contrast, our approach does not rely on gloss to train the model. We combine RGB and keypoint features to extract two distinct latent representations, capturing contextual and spatio-temporal information. Furthermore, Chen et al. [3] use keypoints as heatmaps, which has potential disadvantages. Using keypoints as heatmaps can result in the loss of fine-grained spatial information. When keypoints are represented as heatmaps, the exact spatial coordinates of keypoints may not be preserved with the same level of precision as when using raw keypoints coordinates. This can impact the model’s ability to capture subtle movements and gestures in sign language, which are crucial for accurate translation. In contrast, our method preserves this fine-grained spatial information by using raw keypoint coordinates, potentially leading to more accurate capture and interpretation of sign language gestures.

## 4 Experiments

### 4.1 Datasets

We experimented with SignFormer-GCN on three publicly available datasets: RWTH-PHOENIX-2014T [2], How2Sign [29], and BornilDB v1.0 [30]. A comparative analysis of these datasets is presented in Table 2.

**Table 2:** Comparative analysis of SLT datasets based on language, number of signers, video duration (in hours), vocabulary size (in thousands), and domain attributes.

Dataset	Language	Signers	Duration(h)			Vocabulary(k)			Domain
			Train	Val	Test	Train	Val	Test	
RWTH-PHOENIX-2014T [1]	DGS	9	9.2	0.6	0.7	2	0.9	1	Weather Forecast
How2Sign [29]	ASL	11	69.6	3.9	5.6	15.6	3.2	3.6	Instructional
BornilDB v1.0 [30]	BdSL	3	49.86	5.56	9.86	14.1	3.9	5.4	Not Specified

- RWTH-PHOENIX-2014T [1] is the most used dataset for SLT assessment. The dataset contains German Sign Language (DGS) videos on weather forecasts collected from the German public television station PHOENIX. Along with videos, the dataset has text and gloss annotations in German.
- Compared to the former two datasets, How2Sign [29] is a much larger and much complex dataset. The dataset is made up of American Sign Language(ASL) videos and text annotations of the videos. The dataset is on Instructional Videos on ten different topics.
- BornilDB v1.0 Dataset [30] contains sign language videos of Bangla Sign Language(BdSL) and translated text annotations in Bangla language. Three signers performed the sign language on different topics, but no specific topic is outlined in the dataset paper.

### 4.2 Evaluation protocol

We use reduced BLEU (rBLEU) [50] and BLEU-n [70] scores to measure the performance of our method. BLEU-n score calculates the similarity of the predicted translation to the reference translation. It is compared using n-grams. The precision of each n-gram is calculated by counting the matching n-gram between the reference translation and the predicted translation. The precision is uniformly weighted from 1-grams to n-grams. BLEU score is computed using the following formula:

$$\begin{array}{c} \text{BLEU-n}=\text{BP}\times \text{exp}(\frac{1}{n}\sum_{i=1}^{n}\text{log}\left(p_{i}\right)) \end{array}$$ (12)

$$\begin{array}{c} \text{BP}=\{\begin{array}{cc} 1 & \text{if}\;{\text{MT}}_{\text{length}}>{\text{Ref}}_{\text{length}}\\ \text{exp}(1-\frac{{\text{Ref}}_{\text{length}}}{{\text{MT}}_{\text{length}}}) & \text{if}\;{\text{MT}}_{\text{length}}\leq {\text{Ref}}_{\text{length}} \end{array} \end{array}$$ (13)

Here, utilizing the “Brevity Penalty,” BP, longer sentences are encouraged, and *p*_*i*_ is the precision for n-grams of length i. MT_length_ represents the length of the machine translation, and Ref_length_ denotes the length of the reference translation. Higher BLEU-n scores indicate better translation performance.

rBLEU score is the computation of the BLEU score after removing certain words from the ground truth and prediction. These certain words are generated before training of the model as listed words. Even though listed words are used in the training data, they do not contribute much to the meaning of the sentences. A higher rBLEU score indicates better translation performance.

### 4.3 Implementation details

In the training phase of our model, we employed a batch size of 32 and conducted 250 epochs, each requiring approximately 2.5 minutes for processing on a single NVIDIA GeForce RTX 3090 GPU. We validated after every two epochs of training. Other hyperparameters values are listed in Table 3.

**Table 3:** Optimal hyper-parameters are discovered during experiments.

Hyperparameters	Values
Text Processing	Yes
Batch Size	32
Layers of Encoder	6
Layers of Decoder	3
Attention Heads	4
Learning Rate	1e-3
Feed-Forward Network Dimension	1024
Embedding Dimension	256
Learning Rate Scheduler	cosine

### 4.4 Main results

#### Quantitative results

The effectiveness of our model on the How2Sign dataset [29], compared to the available baseline for this dataset, is illustrated in Table 4.

**Table 4:** Performance comparison of gloss-free translation methods on the How2Sign dataset. (Bold result indicates SignFormer-GCN’s result).

	Validation					Test				
	rBLEU	BLEU-1	BLEU-2	BLEU-3	BLEU-4	rBLEU	BLEU-1	BLEU-2	BLEU-3	BLEU-4
slt_how2sign [31]	2.79	35.2	20.62	13.25	8.89	2.21	34.01	19.3	12.18	8.03
asl_video2text [71]	3.29	35.25	21.03	13.76	9.39	2.56	33.20	19.12	12.10	7.95
**SignFormer-GCN**	**3.97**	**37.37**	**22.18**	**14.45**	**9.9**	**2.96**	**34.91**	**19.98**	**12.72**	**8.53**

In Table 5, we present the comparative results, which include BLEU scores of our model in contrast to the comparison models on the RWTH-PHOENIX-2014T dataset [1]. The results for Joint-SLT [2] were obtained from [72], where they reproduced the results in a gloss-free context. At the same time, we relied on the results originally reported in their respective papers for other models. As illustrated in Table 5, our model significantly improves translation performance while being highly efficient in terms of computational resources. Specifically, our model has only 9.43 million parameters, compared to 115.41 million parameters in the GFSLT-VLP [62] method. Additionally, the training time for our model is approximately 6 hours (200 epochs) on a single NVIDIA GeForce RTX 3090 GPU; in comparison, the GFSLT-VLP [62] approach requires 60 hours across two training phases, using four NVIDIA RTX 3090 GPUs. The GFSLT-VLP [62] approach relies on a symmetric cross-entropy loss similar to the one used in CLIP [73], which is very resource-intensive compared to our method. Regarding inference speed, our model achieves a response time of 0.03–0.034 seconds, while the GFSLT-VLP [62] model takes 0.8–1 second. Our model achieves strong translation performance while being significantly more efficient regarding training time, model size, and inference speed.

**Table 5:** Performance comparison of gloss-free translation methods in the RWTH-PHOENIX-2014T dataset.

Methods	BLEU-1	BLEU-2	BLEU-3	BLEU-4
Conv2d-RNN [1]	27.10	15.61	10.82	8.35
+ Luong Attn. [1] + [74]	29.86	17.52	11.96	9.00
+ Bahdanau Attn. [1] + [75]	32.24	19.03	12.83	9.58
Joint-SLT [2]	30.88	18.57	13.12	10.19
Tokenization-slt [7]	37.22	23.88	17.08	13.25
TSPNet-Sequential [52]	35.65	22.80	16.60	12.97
TSPNet-Joint [52]	36.10	23.12	16.88	13.41
GASLT [72]	39.07	26.74	21.86	15.74
GFSLT-VLP [62]	43.71	33.18	26.11	21.44
**SignFormer-GCN**	**41.19**	**30.89**	**24.23**	**19.75**

In the absence of an established baseline for the BornilDB v1.0 dataset [30], we benchmarked on the dataset in Table 6 to guide future research in BdSL.

**Table 6:** Performance of our gloss-free translation method on the BornilDB v1.0 dataset.

	Validation				Test			
	BLEU-1	BLEU-2	BLEU-3	BLEU-4	BLEU-1	BLEU-2	BLEU-3	BLEU-4
**SignFormer-GCN**	**7.62**	**3.05**	**1.37**	**0.72**	**7.37**	**2.89**	**1.18**	**0.58**

#### Qualitative results

The qualitative results of our methodology on all three datasets are reported in this section.

The qualitative performance of our model on the How2Sign [51] dataset is shown in Table 7. In Table 8, the generated translation of RWTH-PHOENIX-2014T [1] dataset is shown using our best-performing model. As the reference and generated translation are in German, the English translation is also provided for a better understanding of the result. Table 9 displays the translated results of the BornilDB v1 [30] dataset using our most successful model. Given that the reference and generated translations are in Bangla text, English translations are also included in the table.

**Table 7:** Reference and prediction of How2Sign [51] dataset with English translation.

No.	Type	Translation
1	**Reference:**	one more time.
	**Prediction:**	one more time.
2	**Reference:**	the other thing i did was i had a little bit of line out.
	**Prediction:**	another thing i would do is i have a little bit of line out.
4	**Reference:**	and a very natural enhanced lip.
	**Prediction:**	and very natural lips.
5	**Reference:**	just enough to build up some chest strength.
	**Prediction:**	enough to build some chest strength.
6	**Reference:**	going to walk on to our next step.
	**Prediction:**	and we’re going to walk on our next step.
8	**Reference:**	keep your eyes positioned up.
	**Prediction:**	so keep your eye position up.

**Table 8:** Reference and prediction of RWTH-PHOENIX-2014T [1] dataset with English translation.

No.	Type	German (English Translation)
1	**Reference:**	guten abend liebe zuschauer (good evening dear viewers)
	**Prediction:**	guten abend liebe zuschauer (good evening dear viewers)
2	**Reference:**	ihnen noch einen schönen abend (Have a nice evening)
	**Prediction:**	ihnen noch einen schönen abend (Have a nice evening)
3	**Reference:**	hallo und guten abend (Hello and good evening)
	**Prediction:**	hallo und guten abend (Hello and good evening)
4	**Reference:**	und nun die wettervorhersage für morgen dienstag den fünfundzwanzigsten mai (And now the weather forecast for tomorrow, Tuesday May 25th)
	**Prediction:**	und nun die wettervorhersage für morgen dienstag den fünfundzwanzigsten mai (And now the weather forecast for tomorrow, Tuesday May 25th)

**Table 9:** Reference and prediction sentences of BornilDB v1 [30] dataset with English translation.

No.	Type	Bangla (English Translation)
1	**Reference:**	সব কিছু ঠিক আছে তো? (Is everything okay?)
	**Prediction:**	সব ঠিক আছে? (Is everything fine?)
2	**Reference:**	না, আমার মনে হয় না। (No, I don’t think so.)
	**Prediction:**	না, আমার মনে হয় নেই। (No, I don’t feel like it.)
3	**Reference:**	আমি ভালো, বেশ ভালো। (I’m good, pretty good.)
	**Prediction:**	আমি তো অনেক ভাল আছি। (I am very well.)
4	**Reference:**	আমায় ছেড়ে দাও! (Leave me alone!)
	**Prediction:**	আমাকে ছেড়ে দাও। (Let me go.)
5	**Reference:**	ও এসে গেছে! (He has come!)
	**Prediction:**	সে এসে গেল। (He has come.)
6	**Reference:**	সে জানত না? (Didn’t he know?)
	**Prediction:**	সে জানে না? (Does he not know?)

### 4.5 Ablation study

We conducted our ablation investigation on the RWTH-PHOENIX-2014T [1] dataset to enhance the structure and pinpoint the model that performs optimally.

#### Effects of varying the number of STGCN layers

Our experiment investigates the effect of having a different number of STGCN layers. While increasing the number of STGCN layers lets our method acquire a better and more complex representation, it also exposes the method to a higher risk of overfitting. With this objective in mind, we train our method using one to six STGCN layers. As seen in Table 10, the ability of our method to translate gets better with additional STGCN layers initially. However, as we continued to add more STGCN layers, the method overfits the training data, leading to a performance decrease in the test set. For this reason, we use three layers of STGCN in our best-performing model.

**Table 10:** BLEU1, BLEU2, BLEU3 and BLEU4 scores for different STGCN layer configurations.

#Layer	Validation				Test			
	BLEU1	BLEU2	BLEU3	BLEU4	BLEU1	BLEU2	BLEU3	BLEU4
1	40.88	30.40	23.86	19.57	41.02	30.03	23.25	18.87
2	40.99	30.41	23.96	19.72	41.05	30.27	23.62	19.26
3	**40.97**	**30.26**	**23.6**	**19.23**	**41.19**	**30.89**	**24.23**	**19.75**
4	41.40	30.61	23.66	19.19	40.99	30.15	23.45	19.06
5	40.09	29.75	23.27	18.95	41.22	30.45	23.64	19.16

#### Effects of varying the number of transformer encoder and decoder layers

In our next experiment, we explore the impact of employing varying numbers of encoder and decoder layers in the Transformer architecture to find the optimum ones. While increasing the number of layers of encoder and decoder, our method acquires better and more complex representation, and it also exposes the method to a higher risk of overfitting. Considering this objective, we train our method using different encoder and decoder layer combinations. As seen in Table 11, the ability of our method to translate improves with additional encoder and decoder layers. Our model performs best with six layers of encoder layer and three layers of decoder layer.

**Table 11:** BLEU1, BLEU2, BLEU3 and BLEU4 scores for different encoder and decoder layer configurations.

# Encoder Layer	# Decoder Layer	Validation				Test			
		BLEU1	BLEU2	BLEU3	BLEU4	BLEU1	BLEU2	BLEU3	BLEU4
6	3	40.97	**30.26**	**23.6**	**19.23**	**41.19**	**30.89**	**24.23**	**19.75**
2	2	39.15	28.43	21.91	17.7	40.45	29.55	22.78	18.27
3	3	**41.22**	30.29	23.55	19.16	40.69	30.23	23.53	19.1

#### Effects of varying the number of LSTM layers

As seen in Table 12, the ability to translate our method improves initially with additional LSTM layers. But, as we continued to add more LSTM layers, the method overfits the training data, leading to a performance decrease in the test set. For this reason, we use one layer LSTM in our best-performing model.

**Table 12:** BLEU1, BLEU2, BLEU3 and BLEU4 scores for different LSTM layer configurations.

#Layer	Validation				Test			
	BLEU1	BLEU2	BLEU3	BLEU4	BLEU1	BLEU2	BLEU3	BLEU4
1	40.97	30.26	23.6	19.23	**41.19**	**30.89**	**24.23**	**19.75**
2	**41.19**	**30.72**	**23.98**	**19.50**	41.17	30.55	23.75	19.31
3	40.87	30.19	23.32	18.84	41.58	30.59	23.52	18.89

#### Effectiveness of different fusion strategy

To find the best fusion strategy, we experimented with several different ones shown in Table 13. In this work, three different kinds of fusion techniques were used. These fusion solutions combined the encoding of the transformer encoder and the STGCN-LSTM encoder, two distinct architectural streams. Three fusion procedures were used: Fusing with Summation, Fusing with a Linear Layer, and Fusion Using an LSTM Layer. Our technique included Fused with Summation because it outperforms the other strategies.

**Table 13:** rBLEU, BLEU1, BLEU2, BLEU3 and BLEU4 scores for different fusion strategy.

Fusion Strategy	Validation					Test				
	rBLEU	BLEU1	BLEU2	BLEU3	BLEU4	rBLEU	BLEU1	BLEU2	BLEU3	BLEU4
LSTM layer	8.51	38.33	27.62	21.11	16.96	**6.59**	37.96	28.05	21.72	17.66
Linear layer	8.29	40.13	29.04	22.25	17.9	5.97	**40.5**	**29.54**	**22.67**	**18.34**
Summation	**8.76**	**40.64**	**29.8**	**23.27**	**19.01**	6.57	40.28	29.32	22.48	18.02

#### Effectiveness of STGCN-LSTM encoder

We trained our model using and excluding the encoder to find the effectiveness of using the STGCN-LSTM encoder in our architecture. As shown in Table 14, including the STGCN-LSTM encoder in the architecture improves the translation performance.

**Table 14:** rBLEU, BLEU1, BLEU2, BLEU3 and BLEU4 scores for excluding and including STGCN-LSTM encoder.

Fusion Strategy	Validation					Test				
	rBLEU	BLEU1	BLEU2	BLEU3	BLEU4	rBLEU	BLEU1	BLEU2	BLEU3	BLEU4
Excluding STGCN-LSTM	9.56	40.68	29.76	22.95	18.63	6.40	**41.23**	30.39	23.46	18.80
Including STGCN-LSTM	**9.61**	**40.98**	**30.27**	**23.60**	**19.24**	**8.65**	41.19	**30.89**	**24.33**	**19.75**

### 4.6 Limiations

Our approach used transformer architecture, which is resource-intensive due to high computational requirements. Besides, to obtain SLT models for different sign languages, separate training with distinct datasets using the same model is required. This approach limits the model’s ability to translate across multiple languages simultaneously.

## 5 Conclusion and future direction

In this paper, we introduced an approach that uses video and keypoint encoding through the transformer and STGCN architecture to find meaningful contextual and spatiotemporal representation at broader and fine-grained levels to translate sign language videos better. We evaluated our approach on three sign language datasets of three sign languages and reported comparatively good performance for How2Sign [29], RWTH-PHOENIX-2014T [1] and BornilDB v1.0 [30] dataset. In future work, we would like to expand our approach to learn better representation from sign language video by reducing the semantic gap between video encoding, keypoint encoding, and text encoding.
